# Improving the oxidative stability of virgin olive oil using microformulated vitamin‐C

**DOI:** 10.1002/fsn3.2332

**Published:** 2021-05-17

**Authors:** Mahmoud Osanloo, Narjes Jamali, Amene Nematollahi

**Affiliations:** ^1^ Department of Medical Nanotechnology School of Advanced Technologies in Medicine Fasa University of Medical Sciences Fasa Iran; ^2^ Noncommunicable Diseases Research Center Fasa University of Medical Sciences Fasa Iran; ^3^ Department of Food Safety and Hygiene School of Health Fasa University of Medical Sciences Fasa Iran

**Keywords:** ascorbic acid, microemulsion, olive oil, vitamin‐C

## Abstract

This study aims to improve the oxidative stability of olive oil using microformulated vitamin‐C (Vit‐C). The microemulsion containing 10,000 µg/ml Vit‐C with a droplet size of 1,000 ± 68 nm was first prepared. Free radical scavenging of olive oil and olive oil containing blank microemulsion, different amounts of formulated Vit‐C (100–500 µg/ml), and TBHQ (100 and 200 µg/ml as a standard antioxidant) was around 90% during 21 days of incubation at 60°C. The oxidative stability of the mentioned samples during incubation was investigated using the rancimat test, and their quality criteria analysis was studied by peroxide and the acid values. Results showed that the sample's acid value containing 500 µg/ml of Vit‐C did not show significant differences (*p* < .05) with samples containing TBHQ. However, samples containing TBHQ's peroxide value were significant (*p* < .05) lower than samples containing 500 µg/ml of Vit‐C. Furthermore, the induction time of samples containing 500 µg/ml of Vit‐C was significantly (*p* < .05) higher than other treatments during incubation. Thus, the prepared microemulsion could be used as a natural antioxidant in the oil industry instead of harmful synthetic TBHQ.

## Graphical abstract

The clear stable microemulsion of vitamin‐C was prepared using span 80, tween 80, and sesame oil. Samples containing 500 µg/ml of formulated vitamin‐C was effective in controlling virgin olive oil stability. Microemulsion of vitamin‐C could be used as a natural antioxidant in the oil industry instead of harmful synthetic TBHQ.

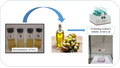

## 1 INTRODUCTION

Recently, the consumption of different kinds of olive oil, including virgin and extra virgin olive oil, has raised considerably due to its appealing taste and aroma, and its nutritional benefits (Abril et al., 2019; Alavi & Golmakani, 2017). Virgin olive oil is achieved from the olive fruit using only physical processes (Alavi & Golmakani, 2017). Its nutritional properties are mainly attributed to its fatty acid composition, essentially because of the high level of oleic acid and the balanced percentage of saturated and polyunsaturated fatty acids (Keramat & Golmakani, 2016). Given that edible vegetable oils generally contain a high amount of unsaturated fatty acids, they are susceptible to oxidation reaction, which is the main reason for oil deterioration and reduced quality (Şahin et al., 2017). This reaction is a complicated phenomenon developed in three radical generation stages, including initiation, propagation, and termination, leading to the formation of peroxide, intermediate peroxides, and stable oxidation products. The oxidation process is stimulated by O_2_, in the existence of initiators such as heat, light, free radicals, and metal ions. This phenomenon could result in the formation of harmful compounds and negatively influence foods' organoleptic properties (Galanakis et al., 2018).

The oil oxidative stability could be increased by applying proper strategies such as keeping away from oxidizing conditions such as light, oxygen, and high temperatures, as well as using antioxidant compounds (either lipophilic or hydrophilic). Butylated hydroxytoluene (BHT), butylated hydroxyanisole (BHA), tertiary butylhydroquinone (TBHQ), and propyl gallate are common synthetic antioxidants in the oil industry to delay oxidation reactions and subsequent generation of toxic components and unpleasantness by reaction with active free radicals (Şahin et al., 2017). They are reducing agents classified in two main categories: A, the primary antioxidants (like BHA, BHT, and TBHQ), which are chain‐breaking agents and act by donating electrons to the oil radicals deactivate them (scavenging free radicals). B, the secondary antioxidants (like ascorbic acid and citric acid) are synergistic antioxidants for enhancing primary antioxidants' activity. They are oxygen scavenger, hydrogen peroxide scavenger, singlet oxygen quencher, and ions metal chelator (Chen et al., 2016; Galanakis et al., 2018; Mohanan et al., 2018; Roschel et al., 2019). As several detrimental consequences for such compounds have been observed, some international monitoring consultants have ascertained limits to the primary synthetic antioxidants' levels allowed in foods. Furthermore, the Codex Alimentarius Commission does not permit synthetic antioxidants into virgin olive oil (Keramat & Golmakani, 2016). Recently, consumers prefer natural compounds worldwide compared with synthetics because the former is recognized as healthy substances (Blasi & Cossignani, 2020). Therefore, enrichment of oils with natural antioxidants has been widely used to retard oil oxidation and its rancidity and improves edible vegetable oils' quality recently (Şahin et al., 2017). Ascorbic acid is a hydrophilic form of vitamin‐C (Vit‐C) and a well‐known secondary antioxidant, which has potent reducing characteristics due to possessing the enediol group (Bodoira et al., 2017). It is an essential water‐soluble vitamin required for normal regulatory metabolism. Its intake has been related to a declining risk of cardiovascular diseases and most cancer types. (Palma‐Rodriguez et al., 2013) Vit‐C is frequently applied to increase the shelf life of numerous food products (Galanakis et al., 2018). Nevertheless, its low solubility in oil causes it unsuitable for oil systems. Consequently, some researchers have examined its esters (mainly palmitate and stearate) in oil systems (Mishra et al., 2021).

The impact of Vit‐C and its derivatives like ascorbyl palmitate and acyl ascorbate on edible oils' oxidative stability was investigated in several studies (Galanakis et al., 2018; Kim et al., 2015; Martínez et al., 2013; Mohanan et al., 2018; Shadyro et al., 2017; Watanabe, 2015; Watanabe et al., 2005). There is no study to assess the effect of a microemulsion of Vit‐C on virgin olive oil's oxidative stability to the best of our knowledge. The present work investigates the effects of different amounts of microformulated Vit‐C on increasing olive oil's oxidative stability. Moreover, the antioxidant activity of microemulsion of Vit‐C was compared with that of TBHQ.

## 2 MATERIALS AND METHODS

Powders of Vit‐C, DPPH (2,2‐diphenyl‐1‐picrylhydrazyl), and TBHQ (tert‐Butyl hydroquinone), as well as absolute ethanol (96%), tween 20, tween 80, span 80, starch, acetic acid, chloroform, thiosulfate sodium, potassium iodine, potassium hydroxide, and phenolphthalein, were purchased from Merck Chemicals, Germany. Virgin olive oil (without any additives) was obtained from Pishgaman Company, Fasa, Iran. Sesame oil was also purchased from a local market, Fasa, Iran.

### 2.1 Preparation of microemulsion of Vit‐C

The spontaneous emulsification approach was used to prepare water in the oil microemulsion of Vit‐C (Zarenezhad et al., 2020). A stock solution of Vit‐C (25% w/v) was prepared using distilled water as the water phase. The water phase (200 µl) was first mixed with different amounts of three surfactants, including span 80, tween 20, and tween 80 at 448 *g* for 10 min. The oil phase (sesame oil) was then added dropwise reached 5,000 µl. The main reason for the selection of sesame oil to prepare Vit‐C microemulsion is related to its composition. In fact, sesame oil is the most resistant vegetable oil to oxidative degradation. It is reported that sesame extracts could be used as alternative antioxidants for the protection of vegetable oils against oxidative deterioration due to phenolic compounds present in sesame extracts like sesamol and sesamolin. (Konsoula & Liakopoulou‐Kyriakides, 2010) The initial quality parameters of sesame oil were determined as follows: The peroxide value, acid value, and induction time were 6.03 meq O_2_/kg, 0.14% oleic acid, and 13.5 hr, respectively. The prepared microemulsions were monitored for 24 hr for any biphasic. The clear and stable microemulsion was selected for size analysis using DLS‐type apparatus (K‐One Nano Ltd, Korea). The mentioned formulation was prepared using 200 µl Vit‐C (25% w/v), 1,150 µl span 80, 1,150 µl tween 80, and 2,500 µl sesame oil. A microemulsion without Vit‐C was also prepared using the same ingredients as the selected microemulsion only without adding Vit‐C, named blank microemulsion.

### 2.2 Enrichment of olive oil with the microemulsion

Different amounts of the selected microemulsion, including 0.4, 0.8, 1.2, 1.6, and 2.0 ml, were added to the proper amount of olive oil reached 40 ml. By adding such mentioned amount, the concentration of Vit‐C was fixed at 100, 200, 300, 400, and 500 µg/ml, respectively. A sample containing 0.4 ml of blank microemulsion was also prepared. Moreover, two samples containing 100 and 200 µg/ml of TBHQ, as the standard antioxidant at no toxic concentration, were prepared as control samples. (Ghaly et al., 2010; Shahabadi et al., 2011) The olive oil samples were placed in an oven at 60°C for 21 days for further investigations, including DPPH assay, thermal‐oxidative stability index (Rancimat test), peroxide value, and acid value.

#### 2.2.1 DPPH assay

DPPH assay was used as described in our previous research to investigate the free radical scavenging effect of no formulated Vit‐C, pure TBHQ (Figure 2), and enrichened olive oil with formulated Vit‐C, blank microemulsion, and TBHQ (Figure 3) (Ghanbariasad & Osanloo, 2020). DPPH stock solution, 3 mM, was prepared by dissolving DPPH powder (MW 394.32 g/mole, 11.83 mg) in 10 ml ethanol. The stock solution was then diluted ten times to prepare the standard solution with a concentration of 0.3 mM. Forty microliter of the samples were added to 96‐well plates separately. After that, a 160 µl/well of DPPH standard solution was added. The treated plates were then incubated away from light for 30 min for performing the reaction.

As Vit‐C is partially soluble in ethanol thus precipitated in plates, each well's supernatant was transferred to another plate to investigate their absorbance (A) at 517 nm using a plate reader (Synergy HTX Multi‐Mode Reader, USA). The antioxidant activity at each concentration was calculated using Equation 1. In each plate, eight wells were considered the control group, filled with 40 µl ethanol and 160 µl DPPH standard solution.

(1) Free radical scavenging(%)=(A control‐A sample/A control)×100

#### 2.2.2 Rancimat test

Oxidative stability assay was performed using the Rancimat test (temperature and gas flow, Metrohm 892, Switzerland). The temperature and the gas flow rate were set at 110°C and 20 L/h, respectively, based on the AOCS (2007) (AOC Society, 1997).

#### 2.2.3 Acid value

The acid value was determined by KOH titration using the AOAC method (2007) (AOC Society, 1997). 2 g of oil sample was dissolved in 50 ml of ethanol (96%). After heating, the solution was titrated with KOH (0.1 N) in the presence of phenolphthalein reagent until being colorless. Acid value (% W/W oleic acid) was calculated by Equation 2:

(2) Acid value=V×56.1×N/M

V is the KOH volume in ml, N is the normality of KOH, and M is the oil sample's weight (mg).

#### 2.2.4 Peroxide value

The peroxide value was measured based on the AOCS (2007) with slight modification (AOC Society, 1997). Briefly, 5 g of the oil sample was dissolved in 30 ml of peroxide solution (containing glacial acetic acid and chloroform (3:2 v/v)). Then, 0.5 ml of saturated potassium iodide (KI) solution was added, and the mixture was shaken for 1 min gently. The mixture is kept in the dark for 1 min, after the addition of 50 ml of distilled water and 0.5 ml of starch solution (1%). The final mixture was titrated with sodium thiosulfate (0.01%) until being colorless. The peroxide value corresponds to the milliequivalent of motivated oxygen per kilogram of oil (meq O_2_/kg), which could oxidize KI with a subsequent release of iodine, which was calculated using Equation 3:

(3) $$\text{Peroxide value}=(V_{s}‐V_{0})\times 1000\times N/M$$

*V_s_* and *V_0_* are the volume of sodium thiosulfate solution consumed for a sample and blank in mL, N is the normality sodium thiosulfate solution, and M is the oil sample weight in g.

### 2.3 Statistical analysis

Statistical analysis was performed using the SPSS version 18 (IBM, USA). Duncan's multiple range test was used to determine significant differences between samples. A *p*‐value < .05 was reflected significant. All experiments were done in triplicate.

## 3 RESULTS AND DISCUSSIONS

### 3.1 Prepared microemulsions

**FIGURE 1:**
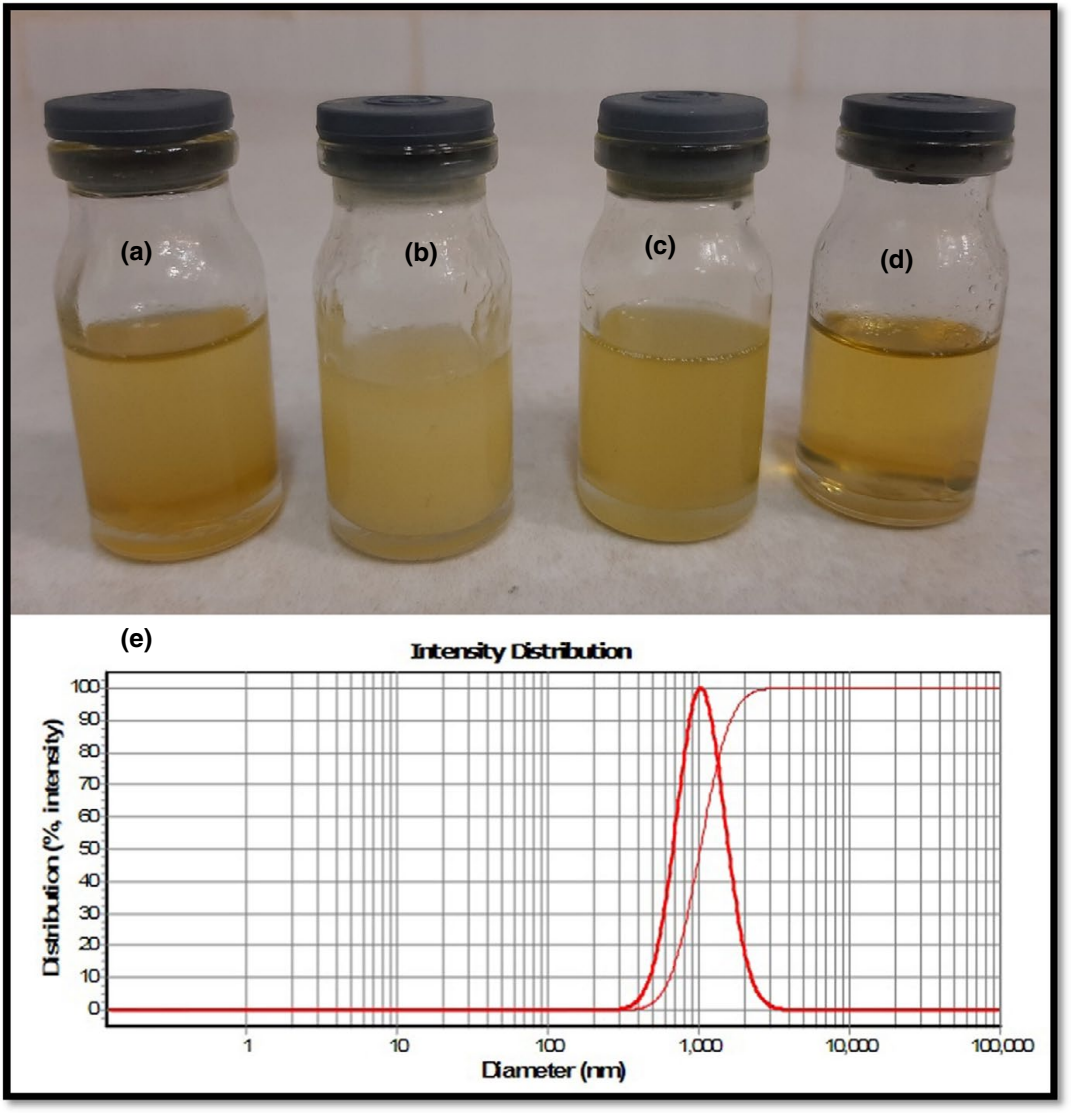
(a), bi‐phased microemulsion (No. 2), (b), Turbid microemulsion (No. 15), (c), Light turbid microemulsion (No. 9 & 19), (d), Clear microemulsion (No. 18), (e), DLS analysis of microemulsion No. 18

Ingredients and visual characteristics of the 19 prepared microemulsions, with a volume of 5,000 µl, are listed in Table 1. Due to the type of microemulsion (water in oil), the highly lipophilic surfactant, that is, SPAN 80, was selected as the main surfactant. Different amounts of SPAN 80, including 500, 1,000, 1,500, 2,000, and 2,500 µl, were screened to determine the appropriate amount, samples 1–5. All samples were bi‐phased after 24 hr; however, samples 2 and 3 were stable around 12 hr (see Figure 1a). It was confirmed that the proper amount of SPAN was in the range of 1,000–1,500 µl. After that, plus to such mentioned amounts of SPAN 80, different amounts (500, 1,000, and 1,500 µl) of two more hydrophilic surfactants, including tween 20 and tween 80, were also screened, samples 6–17. All the samples were biphased, expecting samples 15 and 9; their visual characteristics were turbid and light turbid (see Figure 1b and c). A clear microemulsion was prepared by a slight modification of ingredients; sample 18 (see Figure 1d). DLS analysis of the selected microemulsion (sample 18) with a 1,000 ± 68 nm droplet size is illustrated in Figure 1e. As the peak shows, the size distribution of droplets is narrow. Under the same conditions, clear emulsions contain more uniform droplets than turbid samples, as the emitted laser light (in DLS apparatus) is less scattered. Moreover, biphasic phenomena result from the bigger droplet that precipitates over time due to gravity. The microemulsion was monitored for at least three months; no biphasic, creaming, and precipitation were observed.

**TABLE 1:** Ingredients and visual characteristics of prepared microemulsions

No.	Vit‐C 25% w/v (µl)	Span 80 (µl)	Tween 20 (µl)	Tween 80 (µl)	Sesame Oil (µl)	Results
1	200	500	‐	‐	4,300	BP
2	200	1,000	‐	‐	3,800	BP, 12 hr
3	200	1,500	‐	‐	3,300	BP, 12 hr
4	200	2,000	‐	‐	2,800	BP
5	200	2,500	‐	‐	2,300	BP
6	200	1,000	500	‐	3,300	BP
7	200	1,000	‐	500	3,300	BP
8	200	1,000	1,000	‐	2,800	BP
9	200	1,000	‐	1,000	2,800	Light turbid
10	200	1,000	1,500	‐	2,300	BP
11	200	1,000	‐	1,500	2,300	BP
12	200	1,500	500	‐	2,800	BP
13	200	1,500	‐	500	2,800	BP
14	200	1,500	1,000	‐	2,300	BP
15	200	1,500	‐	1,000	2,300	Turbid
16	200	1,500	1,500	‐	1,800	BP
17	200	1,500	‐	1,500	1,800	BP
18	200	1,150	‐	1,150	2,500	Clear
19	200	1,300	‐	1,300	2,200	Light turbid

### 3.2 Free radical scavenging effect of enrichened samples

**FIGURE 2:**
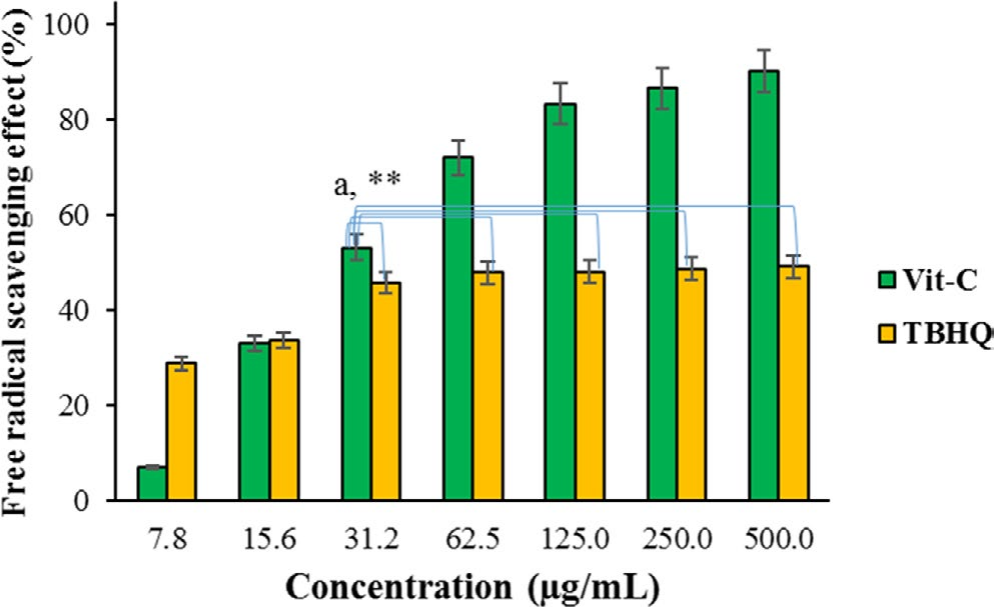
Free radical scavenging effect of no formulated Vit‐C and pure TBHQ at different concentrations. a: Vit‐C at a concentration of 31.2 was significantly (*p* < .05) more potent than TBHQ with the same concentration and higher

The free radical scavenging effect of no formulated Vit‐C and pure TBHQ at different concentrations are depicted in Figure 2. Vit‐C's effect was not significantly changed from 125 to 500 µg/ml (one‐Way ANOVA, *p* > .05); the free radical scavenging rate was constant around 90%. The free radical scavenging activity of TBHQ reached a maximum level (~45%) at a concentration of 31.2 µg/ml and higher points; this amount was significantly even lower than Vit‐C at a concentration of 31.2 µg/ml (independent sample *t*‐test, *p* < .05). Noted, DPPH assay is based on reducing stable free radicals; DPPH is reduced to DPPHH by accepting an odd electron (Herald et al., 2012; Shekhar & Anju, 2014).

**FIGURE 3:**
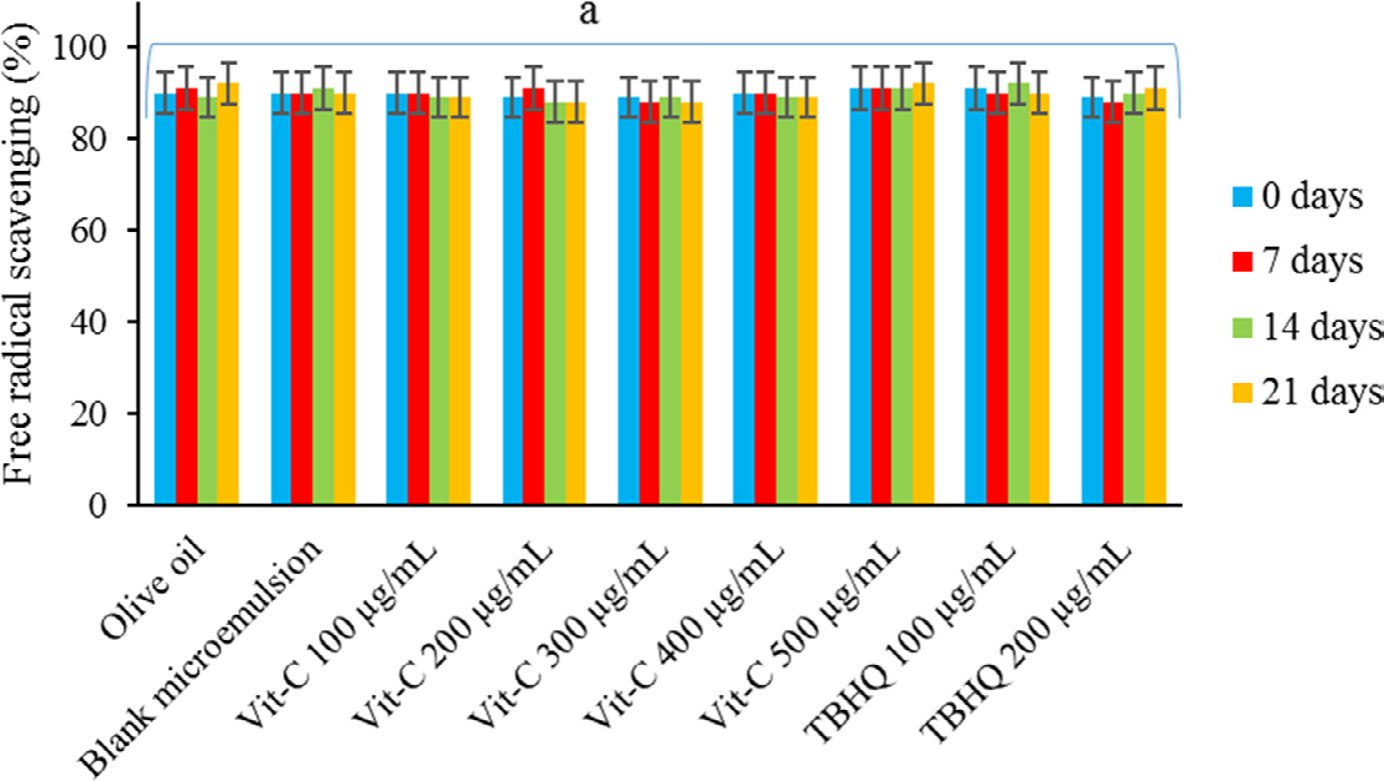
Free radical scavenging effects of olive oil and olive oil containing blank microemulsion, formulated vit‐C (100–500 µg/ml), and TBHQ (100–200 µg/ml). a: there was no significant difference (*p* > .05) were observed in free radical scavenging of samples incubated at 60°C for different periods

Furthermore, free radical scavenging of olive oil, olive oil containing 100–500 µg/ml formulated Vit‐C, olive oil containing blank microemulsion, and olive oil containing 100 and 200 µg/ml of TBHQ after different periods of incubation at 60°C are illustrated in Figure 3. Interestingly, no significant differences (*p* > .05) were observed among free radical scavenging of all samples even after incubations at different incubation periods (0, 7, 14, and 21 days); they were around 90%. Increasing free radical scavenging of samples containing TBHQ is related to natural antioxidants in olive oil and sesame oil; they contain powerful antioxidants such as phenolic compounds (Konsoula & Liakopoulou‐Kyriakides, 2010; Lanza & Ninfali, 2020; Vissers et al., 2004).

TBHQ, BHA, and BHT are the most widely used antioxidants in the food and oil industries (Ni et al., 2000). However, TBHQ only could be added to food products up to 0.02% (Ghaly et al., 2010; Shahabadi et al., 2011), and it is suspected to having adverse effects on health, such as precursors to stomach tumors and damage to DNA (Eskandani et al., 2014; Kashanian & Dolatabadi, 2009). As the results of this study showed, TBHQ possesses a moderate free radical scavenging effect; the natural antioxidants in olive oil seem to be sufficient to neutralize free radicals.

Vit‐C is commonly added to fruit juices as a potent preservative and antioxidant supplement; however, it could not be used in oils due to its hydrophilicity. (Shaikh & Deshmukh, 2019; Tenore et al., 2012) In the current study, this application was made possible by formulating Vit‐C. Moreover, by enriching olive oil with Vit‐C, a stronger antioxidant is used, and the benefits of Vit‐C in the body could be exploited. In addition to free radical scavenging, Vit‐C could manage high blood pressure, prevent iron deficiency, and reduce heart disease risk (Chambial et al., 2013; Jacob & Sotoudeh, 2002).

### 3.3 Thermal oxidative stability during incubation

In this stage, oxidative stability of olive oil, olive oil containing 100–500 µg/ml formulated Vit‐C, olive oil containing blank microemulsion, and olive oil containing 100 and 200 µg/ml TBHQ were examined.

Oxidative stability is a prominent procedure for assessing the oils’ quality because it gives an effective evaluation of their proneness to oxidative degradation, the principal reason for their change. It is worthy to note that the oxidative progression depends on several factors, including the presence of light and oxygen, temperature, fatty acid profile, and the type and level of the antioxidant and pro‐oxidant components. For calculating the virgin olive oil resistance to the oxidation process during the storage period, the rancimat test is one of the most commonly used methods. It is performed in a commercially accessible apparatus, the method is standardized, and outcomes are acquired quickly (Mateos et al., 2005). Rancimat is an accelerated technique for estimating the oxidative stability of oils, representing the duration of shelf‐life. This method is based on the induction time, a measurement of the time extent for initiating an oil oxidative degradation. The oil type, degree of unsaturation, and antioxidants’ effect on induction time should be evaluated (Şahin et al., 2017). The changes in rancimat induction periods of all the samples during the 21 days of incubation at 60°C are presented in Table 2. The induction period of all samples decreased significantly (*p* < .05) during the incubation period from 7.42–8.53 to 1.4–3.63 hr. Obviously, increasing the concentration of Vit‐C from 100 to 500 µg/ml resulted in a significant increase (*p* < .05) in thermal stability of olive oil in all days of incubation, which reached 1.4, 1.88, 2.28, 2.90, and 3.63 hr, respectively, on day 21. As presented in Table 2, the thermal stability of samples containing 200 µg/ml TBHQ was also significantly (*p* < .05) higher than those containing 100 µg/ml of this synthetic antioxidant in all incubation times, which is in good agreement with Mohammadi et al. (2016) study. (Mohammadi et al., 2016) Koprivnjak et al. (2008) also investigated the effect of adding lecithin (as a natural antioxidant) on the oxidative stability of the virgin olive oil by the Rancimat method; it was found a positive correlation between antioxidant concentration and induction time. They reported a higher concentration of lecithin had a noticeable auto‐oxidation inhibition activity in virgin olive oil (Koprivnjak et al., 2008). However, in Özkan and Özkan (2017) study, no significant difference was observed among the stability of virgin olive oils containing 600 and 1,200 ppm herb extracts (Özkan & Özcan, 2017).

**TABLE 2:** Induction time (Rancimat analysis) of olive oil samples during 21 days incubation at 60°C (h)

Treatments*	Incubation time (day)			
	0	7	14	21
Vit‐C 100 µg/ml	7.60 ± 0.20^aA**^	2.91 ± 0.18^aB^	1.40 ± 0.02^aC^	1.40 ± 0.08^aC^
Vit‐C 200 µg/ml	7.50 ± 0.01^bA^	4.40 ± 0.09^bB^	3.72 ± 0.32^bC^	1.88 ± 0.06^bD^
Vit‐C 300 µg/ml	7.42 ± 0.03^cA^	6.60 ± 0.01^cB^	5.46 ± 0.01^cC^	2.28 ± 0.02^cD^
Vit‐C 400 µg/ml	7.70 ± 0.02^dA^	7.28 ± 0.12^dB^	4.42 ± 0.01^dC^	2.90 ± 0.02^dD^
Vit‐C 500 µg/ml	8.53 ± 0.05^eA^	8.31 ± 0.07^eB^	7.53 ± 0.03^eC^	3.63 ± 0.09^eD^
Olive oil	8.10 ± 0.10^fA^	5.45 ± 0.02^fB^	2.89 ± 0.04^fC^	1.87 ± 0.11^bD^
Blank microemulsion	8.23 ± 0.02^gA^	5.16 ± 0.01^gB^	2.49 ± 0.02^gC^	1.58 ± 0.20^fD^
TBHQ 100 µg/ml	8.10 ± 0.06^fA^	4.50 ± 0.01^hB^	3.31 ± 0.16^hC^	1.91 ± 0.01^bD^
TBHQ 200 µg/ml	7.90 ± 0.09^gA^	4.82 ± 0.03^iB^	4.01 ± 0.10^iC^	3.54 ± 0.02^gD^

* Olive oil containing 100–500 µg/ml vit‐C, olive oil containing blank microemulsion, control olive oil, and olive oil containing 100 and 200 µg/ml of TBHQ.

** Each value in the table is the mean ± standard deviation (*SD*) of three trials. Different lower and upper letters in each column and row, respectively, indicate a statistically significant difference (*p* < .05).

In the current study, the highest thermal stability was related to the sample containing 500 µg/ml of formulated Vit‐C. This sample's induction period was reduced significantly (*p* < .05) from 8.53 (day 0) to 3. 63 hr (day 21). We observed that the thermal stability of olive oil samples enriched with 300–500 µg/ml of Vit‐C was significantly (*p* < .05) higher than control samples (without any additives), and even those samples containing TBHQ (100 and 200 µg/ml). This was verified by observing the lowest thermal stability for the oil samples containing 100 µg/ml Vit‐C followed by the control sample, which was 1.4 and 1.58 hr, respectively, at the end of incubation (Table 2). Higher induction time means it will take a prolonged time to create volatile oxidation products and better the oil's oxidative stability. (Mohanan et al., 2018) For example, the significant (*p* < .05) higher induction time of samples containing 500 µg/ml Vit‐C than that in samples containing 100 and 200 µg/ml TBHQ indicates its ability to increase the oxidative stability of virgin olive oil, which complies with Mohanan et al., (2018) and Watanabe et al., (2005) studies.

### 3.4 Quality criteria analyze during incubation

The selected parameters, including peroxide value and acid value, were screened to evaluate the quality of olive oil samples during incubation.

Table 3 shows the acid value of olive oil samples during the 21 days of the heat incubation period. The initial acid value varied from 1.5% to 1.96% oleic acid, and the values increased significantly (*p* < .05) in all samples throughout the heat incubation time, indicating lowering oil quality. The acid value reached 5.01% oleic acid on day 21 for the control olive oil (whiteout any additives) samples, which are significantly (*p* < .05) higher than other samples in all incubation times. According to the Iranian National Standardization Organization, the maximum limit for virgin olive oil's acid value is 2% oleic acid. (ISIRI 2012) Our results showed that unacceptable acid value was found in control, blank microemulsion samples, and samples containing 100–200 µg/ml formulated Vit‐C from day 7 of incubation, which was 2.81%, 3.35%, and 2.19%, 2.81%, and 2.81% oleic acid, respectively. The acid value of sample containing 400 µg/ml formulated Vit‐C exceeded maxim limit (2% oleic acid) from day 14, while this value for samples containing 500 µg/ml formulated Vit‐C, 100 and 200 µg/ml TBHQ surpassed the maximum limit from day 21 which were 2.24%, 2.23% and 2.23% oleic acid, respectively.

**TABLE 3:** The acid value of olive oil samples during 21 days incubation at 60°C (% oleic acid)

Treatments*	Incubation time (day)			
	0	7	14	21
Vit‐C 100 µg/ml	1.93 ± 0.01^aA^	2.81 ± 0.04^aB^	4.47 ± 0.06^aC^	4.72 ± 0.01^aD^
Vit‐C 200 µg/ml	1.60 ± 0.01^bA^	2.81 ± 0.02^aB^	3.35 ± 0.09^bC^	4.18 ± 0.01^bD^
Vit‐C 300 µg/ml	1.69 ± 0.04^cA^	2.19 ± 0.01^bB^	2.22 ± 0.01^cB^	3.37 ± 0.04^cC^
Vit‐C 400 µg/ml	1.52 ± 0.01^dA^	1.68 ± 0.01^cB^	2.10 ± 0.10^dC^	2.81 ± 0.12^dD^
Vit‐C 500 µg/ml	1.52 ± 0.03^dA^	1.67 ± 0.03^cB^	1.98 ± 0.05^eC^	2.24 ± 0.02^eD^
Olive oil	1.96 ± 0.02^aA^	3.35 ± 0.05^dB^	4.21 ± 0.09^fC^	4.21 ± 0.21^bC^
Blank microemulsion	1.40 ± 0.04^eA^	2.81 ± 0.06^aB^	4.21 ± 0.10^fC^	5.01 ± 0.30^fD^
TBHQ 100 µg/ml	1.56 ± 0.01^dA^	1.96 ± 0.04^eB^	1.96 ± 0.02^eB^	2.23 ± 0.01^eC^
TBHQ 200 µg/ml	1.54 ± 0.05^bdA^	1.95 ± 0.01^eB^	1.95 ± 0.02^eB^	2.23 ± 0.03^eC^

* Olive oil containing 100–500 µg/ml vit‐C, olive oil containing blank microemulsion, control olive oil, and olive oil containing 100 and 200 µg/ml of TBHQ.

** Each value in the table is the mean ± standard deviation (*SD*) of three trials. Different lower and upper letters in each column and row, respectively, indicate a statistically significant difference (*p* < .05).

Regarding the obtained results, as the percent of Vit‐C increased, the acid value decreased significantly (*p* < .05) in all days of incubation, especially on days 14 and 21. For example, the acid value of olive oil samples containing 500 µg/ml formulated Vit‐C was significantly (*p* < .05) lower than other samples containing a lower concentration of formulated Vit‐C (100–400 µg/ml) on days 14 and 21 of the incubation period. Free fatty acids level is increased throughout the incubation period; this multiplication is considerably related to oil degradation. With the degradation of triglycerides and a further increase of free fatty acid concentrations, the oxidation process in vegetable oils is progressed, and thus, their shelf life is declined (Tavakoli et al., 2017). As shown in Table 3, the acid value of samples containing 500 µg/ml did not show significant differences (*p* > .05) with samples containing 100 and 200 µg/ml of TBHQ. Thus, it is worthy to note that the addition of 500 µg/ml of formulated Vit‐C was sufficient to postpone producing free fatty acids in olive oil similar to synthetic antioxidant agents, that is, TBHQ.

When it comes to food safety and quality, the restriction of oil autoxidation in food products is crucial to preclude foods from deterioration and ensure human health. Given that the peroxide value is an indicator of the oils' primary oxidation state, this value plays a critical role in quality control determinations for edible oils. This value represents the level of first oxidation compounds, including hydroperoxides, which are changeable substances and can decompose to generate oxygenated components with low molecular weight, including free fatty acids, alcohols, aldehydes, and ketones, finally causing rancidity. (Mohammadi et al., 2016) Thus, this value declines once secondary oxidation products such as alcohols, aldehydes, and ketones form. An increase in peroxide value indicates the increase in the generation of primary oxidation products in oil (Mohanan et al., 2018). From Table 4, the peroxide value of all samples increased significantly (*p* < .05) during the 21 days of incubation. Increasing samples containing 100, 300, and 500 µg/ml formulated Vit‐C and 100, and 200 µg/ml TBHQ are significantly (*p* < .05) lower than others that means degradation of peroxides is quicker than their formation (Galanakis et al., 2018). Noted, the peroxide value of control olive oil and olive oil containing blank microemulsion reached 36.80 and 36.72 meq O_2_/kg oil after 21 days of incubation, which did not show significant differences (*p* > .05). While the peroxide value for olive oil samples containing 200, 300, 400, and 500 µg/ml Vit‐C were 26.00, 23.00, 23.90, and 19.95 meq O_2_/kg oil, respectively, which showed significant differences (*p* < .05). The peroxide value for those samples consisting of 100 and 200 µg/ml TBHQ reached a maximum of 21.4 and 20.57 meq O_2_/kg oil, respectively, after 14 days of incubation at 60°C. It is worthy to note that the generation and propagation of these peroxides are probably hampered by the free radical scavenging activity of Vit‐C and its synergistic effects with natural polyphenolic antioxidants present in olive oil (Mohanan et al., 2018). Özkan and Özkan (2017) indicated that herb extracts could be used as natural antioxidants to preserve virgin olive oil stored for 28 days at 60°C. They found the highest amount of peroxide value in BHA‐containing oils, which were in order of 2.98–29.64 meq O_2_/kg at the end of storage. However, the peroxide values of oil samples containing BHT were similar to those containing herb extracts until the 21st day of study. The acid value of oil containing 1,200 mg/L of herb extract was established as the lowest amount compared with other samples. Similar to our results, herb extracts have a good ability to decrease the rate of peroxide formation in olive oil compared with synthetic antioxidants like BHA and BHT (Özkan & Özcan, 2017).

**TABLE 4:** Peroxide value of olive oil samples during 21 days incubation at 60°C (meq O_2_/kg)

Treatments*	Incubation time (day)			
	0	7	14	21
Vit‐C 100 µg/ml	10.02 ± 0.12^aA^	18.00 ± 0.12^aB^	34.60 ± 0.20^aC^	28.00 ± 0.11^aD^
Vit‐C 200 µg/ml	9.49 ± 0.02^bA^	17.80 ± 0.11^bB^	25.34 ± 0.34^bC^	26.00 ± 0.32^bD^
Vit‐C 300 µg/ml	10.97 ± 0.05^cA^	17.10 ± 0.02^cB^	25.00 ± 0.33^cC^	23.00 ± 0.14^cD^
Vit‐C 400 µg/ml	10.98 ± 0.01^cA^	15.40 ± 0.17^dB^	22.20 ± 0.09^dC^	23.90 ± 0.60^dD^
Vit‐C 500 µg/ml	10.09 ± 0.22^aA^	12.37 ± 0.42^eB^	21.95 ± 0.41^eC^	19.95 ± 0.04^eD^
Olive oil	10.21 ± 0.01^dA^	17.20 ± 0.20^fB^	34.40 ± 0.51^fC^	36.72 ± 0.17^fD^
Blank microemulsion	10.00 ± 0.27^aA^	18.80 ± 0.11^gB^	35.00 ± 0.17^gC^	36.80 ± 0.64^fD^
TBHQ 100 µg/ml	10.62 ± 0.17^eA^	11.00 ± 0.21^hB^	21.40 ± 0.28^hC^	19.78 ± 0.27^gD^
TBHQ 200 µg/ml	10.35 ± 0.24^fA^	10.57 ± 0.13^iB^	20.57 ± 0.08^iC^	19.18 ± 0.14^hD^

* Olive oil containing 100–500 µg/ml vit‐C, olive oil containing blank microemulsion, control olive oil, and olive oil containing 100 and 200 µg/ml of TBHQ.

** Each value in the table is the mean ± standard deviation (*SD*) of three trials. Different lower and upper letters in each column and row, respectively, indicate a statistically significant difference (*p* < .05).

According to the Iranian National Standardization Organization, the maximum limit for peroxide value of virgin olive oil is 20 meq O_2_/kg oil (ISIRI 2012) Our results showed that unacceptable peroxide values were found in all samples from day 14 of incubation. In this regard, the peroxide value of samples containing 500 µg/ml formulated Vit‐C as well as 100 and 200 µg/ml TBHQ exceeded the maximum limit (20 meq O_2_/kg oil) from day 14, which were 21.95, 21.40, and 20.57 meq O_2_/kg oil, respectively.

Our results depicted a high concentration of Vit‐C (500 µg/ml) could be considered a natural antioxidant instead of harmful synthetic ones like TBHQ in vegetable oil preservation. Galanakis et al., (2018) also reported that the addition of Vit‐C in the form of ascorbyl palmitate (1,000 and 3,000 µg/ml) sustained the peroxide value of olive oil in comparison with α‐tocopherol, which is in good agreement with our study. The addition of 500 µg/ml formulated Vit‐C could be attributed to its synergistic activity with the polyphenols presented inherently in vegetable oils, decreasing the peroxy radicals. The ability of Vit‐C and polyphenols to retard the first step of lipid autoxidation is extremely critical. If the oxidation process speeds up, it is very challenging to postpone it. (Galanakis et al., 2018) In this regard, Vit‐C can regenerate the tocopherols inherently present in the olive oil, which could decrease lipid oxidation (Mohanan et al., 2018) and is justified in Bodoira et al.’s, 2017) investigation by application of Vit‐C in the form of ascorbyl palmitate for improvement of oil shelf life. Vit‐C has an enediol group in its structure, which shows reducing capacity. In this regard, Vit‐C can efficiently postpone the generation of lipid oxidation products in edible oils and model systems (Kim et al., 2015).

Furthermore, it is reported that Vit‐C has several antioxidants activities like reaction with free radicals, as singlet oxygen quencher (Martínez et al., 2013). Mohanan et al., (2018) also found that Vit‐C with intermediate polarity in the form of ascorbyl palmitate was better in maintaining oxidative stability of flaxseed oil than hydrophilic (tannic acid) and hydrophobic (α‐tocopherol) antioxidants due to higher radical scavenging and iron and copper chelating activities. Overall, Vit‐C could be used as a potent antioxidant due to its capability to donate hydrogen atoms to quench free radicals. After scavenging, Vit‐C converts into dehydroascorbic acid, recovering activity following receiving hydrogen atoms (Abbas et al., 2012). Regarding the current study results, Vit‐C probably seems to have a synergistic effect in association with some primary antioxidant (phenolic) compounds present in olive oil since ascorbic acid could act as secondary antioxidants. However, there is a need to further research the synergistic correlation of natural antioxidants present in olive oil and Vit‐C during storage and heat food processing.

## 4 CONCLUSION

The clear microemulsion of Vit‐C was first prepared using sesame oil, tween 80, and span 80. The samples' induction time containing 500 µg/ml of formulated Vit‐C was significantly (*p* < .05) higher than samples containing 100 and 200 µg/ml TBHQ indicating its ability to increase the oxidative stability of virgin olive oil. Furthermore, the quality criteria (acid and peroxide value) of olive oil samples containing 500 µg/ml formulated Vit‐C was approximately similar to those containing 200 µg/ml TBHQ. Thus, the prepared Vit‐C's microemulsion could be introduced as a potent natural antioxidant and even substituted with a broadly used synthetic antioxidant, TBHQ.

## CONFLICTS OF INTEREST

No researchers have a conflict of interest in this study.

## AUTHOR CONTRIBUTION

**Mahmood Osanloo:** Data curation (equal); Methodology (equal); Project administration (equal); Supervision (equal); Writing‐original draft (equal); Writing‐review & editing (lead). **Narjes Jamali:** Data curation (equal); Formal analysis (lead). **Amene Nematollahi:** Data curation (equal); Methodology (equal); Project administration (equal); Supervision (lead); Writing‐original draft (equal); Writing‐review & editing (equal).

## Data Availability

The data that support the findings of this study are available on request from the corresponding author. The data are not publicly available due to privacy or ethical restrictions.
